# The impact of proliferation-migration tradeoffs on phenotypic evolution in cancer

**DOI:** 10.1038/s41598-019-39636-x

**Published:** 2019-02-20

**Authors:** Jill A. Gallaher, Joel S. Brown, Alexander R. A. Anderson

**Affiliations:** Department of Integrated Mathematical Oncology, H. Lee Moffitt Cancer Center, Tampa, FL USA

## Abstract

Tumors are not static masses of cells but dynamic ecosystems where cancer cells experience constant turnover and evolve fitness-enhancing phenotypes. Selection for different phenotypes may vary with (1) the tumor niche (edge or core), (2) cell turnover rates, (3) the nature of the tradeoff between traits, and (4) whether deaths occur in response to demographic or environmental stochasticity. Using a spatially-explicit agent-based model, we observe how two traits (proliferation rate and migration speed) evolve under different tradeoff conditions with different turnover rates. Migration rate is favored over proliferation at the tumor**’**s edge and vice-versa for the interior. Increasing cell turnover rates slightly slows tumor growth but accelerates the rate of evolution for both proliferation and migration. The absence of a tradeoff favors ever higher values for proliferation and migration, while a convex tradeoff tends to favor proliferation, often promoting the coexistence of a generalist and specialist phenotype. A concave tradeoff favors migration at low death rates, but switches to proliferation at higher death rates. Mortality via demographic stochasticity favors proliferation, and environmental stochasticity favors migration. While all of these diverse factors contribute to the ecology, heterogeneity, and evolution of a tumor, their effects may be predictable and empirically accessible.

## Introduction

**Figure 1:**
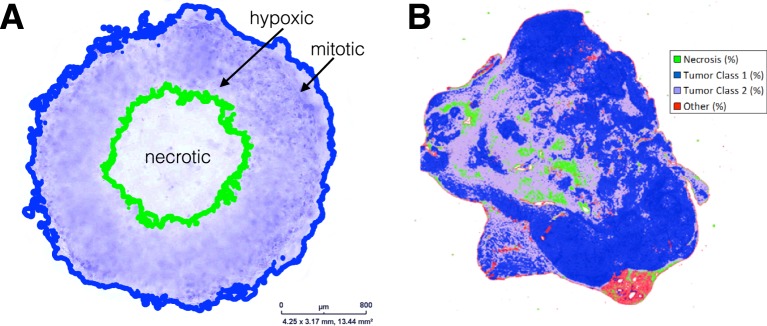
Tumor anatomy in spheroid models and human tumors. (**A**) Tumor spheroid model. Edge detection algorithm finds inner necrotic (green) and outer proliferating (blue) edges. Image provided by Mehdi Damaghi. (**B**) Digital pathology uses pattern recognition on histological sample from actual tumor. The proliferating, hypoxic and necrotic regions have the same broad structure but are more intermixed. Image provided by Mark Lloyd.

Tumors are thought to consist of 3 major populations of cells: actively dividing, quiescent and necrotic. Under idealized environments, such as the experimental system of spheroids^1^, a fast growing tumor becomes dense and quickly outgrows the supply of oxygen and nutrients. This gives rise to a layered tumor anatomy that consists of concentric regions encompassing the 3 populations (e.g. Fig. 1A). In real tumors, the geometry of these regions appears far more irregular and disordered (e.g. Fig. 1B), reflecting a more complex and dynamic environment. Regardless, it is a tempting simplification to view the tumor edge as the place where tumor cells primarily divide rather than die, the interior as generally quiescent with few births and deaths, and the necrotic zone where tumor cells mostly die.

Such a perspective has led to models of tumor growth and evolution where tumor cells expand to occupy space, either explicitly^2–11^ or implicitly^12–15^, as different clonal lineages proliferate and expand at different rates. When these models include evolution, one can determine the properties of tumor cells that are favored by natural selection. Such is the case for models that examine the joint evolution of proliferation and migration^2,3,7^. However, in the absence of cell turnover, such models can only show changes in the frequency of different clonal lineages while the replacement of less successful lineages by more successful ones is ignored.

In reality, the turnover of tumor cells via proliferation and cell death occurs constantly throughout the entirety of the tumor. Turnover rates may be high, as high as every 10 days for the interior of breast cancer tumors. A tumor that looks static with an unchanging volume might actually be very dynamic as proliferation and apoptosis occur in parallel throughout a tumor. High but balanced proliferation and death rates have been measured in some cancers^16–19^. Furthermore, stimulatory factors from dying cells can cause compensatory proliferation of surviving cells^20^, and an increased proliferation along with an increased death rate may suggest a more aggressive disease^18,19^.

High turnover rates facilitate evolution by natural selection^21^. This “struggle for existence” is seen in all organisms, and in cancer the cells have the capacity to produce more offspring than can possibly survive. Competition for space and resources limits cancer cell densities and population sizes. Limits to growth and cell turnover should select for genes and traits associated with proliferation rates and movement. All else equal, the cancer cell lineage with a higher proliferation rate will outcompete and replace one with a slower proliferation rate. However, higher proliferation rates will cause local crowding, limitations on resources, and other unfavorable conditions. Movement and migration away from such crowding should be favored. Even random migration can be favored by natural selection as a means of avoiding over-crowding^22^. Such migration can be particularly favorable at the edge of the tumor, but even in the interior of a tumor, migration may move cells from more to less dense locales.

Many mutation models of cancer progression allow for unconstrained phenotypic improvement^2,3,5^ or infer increased fitness through the number of passenger/driver mutations^23,24^. Indeed, if both proliferation and migration enhance the fitness of cancer cells, then natural selection should favor higher rates for both^3^. Such selection will continue to improve proliferation and migration rates simultaneously until a point is reached where there are tradeoffs^25–27^. To improve proliferation rates further necessarily means sacrificing migration and vice-versa^28–30^. In his seminal book on evolution of changing environments, Levins (1967) proposed that the shape of the tradeoff curve should influence the evolutionary outcome^31^. A convex curve may favor a single population with a generalist phenotype whereas a concave curve may favor the coexistence of two specialist populations. Additionally, the specific shape of the tradeoff curve can significantly affect the evolutionary trajectory towards this curve^32^.

The pattern of cancer cell mortality across a tumor may represent just *demographic stochasticity* or it may include *environmental stochasticity*^33^. The former happens when cell death is random and exhibits little temporal or spatial autocorrelations. Such patterns of mortality open up numerous but small opportunities for cell replacement. Environmental stochasticity happens when the sudden absence of nutrients or the accumulation of toxins causes wholesale death of the cells in some region of the tumor. This pattern of cell mortality creates fewer but much larger spaces for cell replacement. When regions are subject to catastrophic death (e.g., large or small temporary regions of necrosis) the distinctiveness of edge versus interior regions of a tumor are obscured, and the evolution of different combinations of proliferation and migration rates may be favored. Strictly demographic stochasticity should favor proliferation over migration and vice-versa for environmental stochasticity within the tumor.

In what follows, we develop a spatially explicit agent-based model of tumor growth that includes cell turnover at both the edge and the interior of the tumor. We use this model to explore the joint evolution of proliferation and migration rates by cancer cells in response to: (1) rates of cell turnover, (2) different shapes of the tradeoff curve, (3) and different mortality regimes.

## The Model

**Figure 2:**
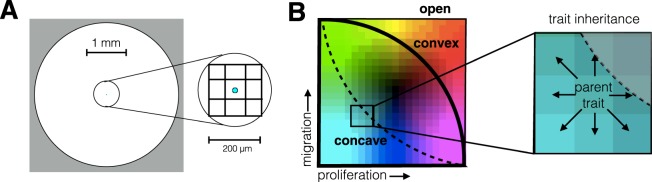
Mathematical model details. (**A**) A single cell with the smallest proliferation and migration rates centered in a 4-mm radial boundary initializes the simulation. The cell diameter is 20 μm, and its area of interaction is defined by a 200 × 200 micron neighborhood (more detail can be found in the Methods section). (**B**) Imposing tradeoffs by bounding the phenotype space. When the whole space is open (thin solid line), all phenotypes are allowed. The convex (thick solid line) and concave (dashed line) bound the space as shown. The set of evolutionarily feasible traits lies within (fitness set) and on the tradeoff line (active edge). The trait value of a daughter cell can mutate up to one unit in any direction as long as it stays within the bounded region.

Using an off-lattice agent-based model, we investigated how 2 traits (proliferation and migration) will evolve in response to space limitation and the continual turnover of cells. Initially, we start with a single cell in the center of a 2D circular domain with the least aggressive phenotype: a long cell cycle time and a slow migration speed. Figure 2A shows the 4 mm diameter circular space available to the tumor and its starting location. Density-dependence and limits to population growth comes from local crowding, similar to the methods presented in Gallaher *et al*.^3^. When a cell is completely surrounded by neighbors, we assume that it can neither move nor divide. Upon division, each daughter cell’s trait may change in one of three ways: it can inherit the same trait as the original cell, or via mutation, its trait values for migration or proliferation rate can increase or decrease by a small value, so long as its trait values stay within the boundaries of what is evolutionarily feasible. Figure 2B shows the trait space with respect to proliferation and migration, and how an open, convex, or concave boundary in the trait space eliminates possible trait combinations. More details on the model specifics can be found in the Methods section.

## Results

In the following, we used the model described in the previous section to investigate how proliferation-migration tradeoffs and cell turnover affect the evolution of phenotypes over time.

### Imposing a go-or-grow tradeoff selects for migration during growth

Evolutionarily, we placed limits on the set of feasible combinations of migration and proliferation. The boundary of this set represents the tradeoff between the two traits. In our simulations, we considered three forms of the tradeoff: open, convex, and concave boundary conditions (Fig. 2B). Under an open tradeoff, each trait can achieve a maximum value independent of the value of the other trait (no tradeoffs). Under a convex (or concave) tradeoff, the maximum feasible values for migration and proliferation occur along a curve that bows outwards (or inwards). Regardless of the shape of the boundary, natural selection should favor cancer cells with ever greater migration and proliferation rates until reaching the boundary edge. However, the shape of the tradeoff may influence both the evolutionary trajectory of the cancer cells, their evolutionary endpoint, and the diversity or variance of trait values among the cancer cells.

**Figure 3:**
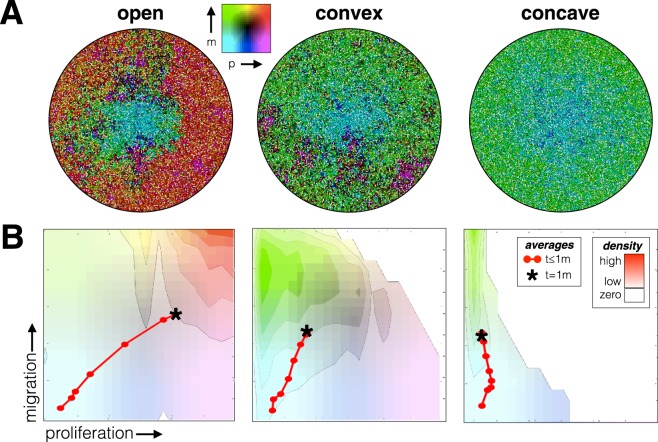
Joint evolution of migration and proliferation as influenced by three different tradeoff boundaries: open, convex, and concave. (**A**) The spatial layout and (**B**) the frequency of trait combinations is shown for a single representative simulation for each case, where the red points and line mark the average trait values every 5 days. The background colors correspond to the density of cell traits after reaching capacity; Brightly colored areas correspond to high densities, and the completely white area contains no cells. Replicate simulation runs are shown in Fig. S1 (top).

Ecologically, we first considered the case where there is no cell mortality. In this case, the population of cells will divide and migrate until the space is filled completely (see Fig. 3A for the spatial layout). In the absence of death, we see rings of cells with different phenotypes within the tumor. While natural selection favors cells with greater trait values, these trait values can only arise through successive cell divisions. The least aggressive cells, those with the lowest trait values, form the core (cyan color). Towards the outer edges, cells with more aggressive traits predominate at the periphery. Cells that mutate with higher proliferation rates can increase in frequency where space permits, and cells that mutate with higher migration rates can move into empty spaces where longer runs of proliferation are possible. Even as the whole population evolves, each step in this evolution leaves tree ring like layers in the tumor. With no cell death, the entire historical record in space and time is preserved.

Once the space has filled, the distribution of cancer cell phenotypes can be seen in Fig. 3B in the form of a density map. Color intensities correspond to the relative frequency of phenotypes where white indicates an absence of cells with that phenotype. As expected, when the tradeoff boundary between migration and proliferation is open, the most frequent phenotypes exhibit both fast proliferation and fast migration. However, we did observe some variation between replicate simulation runs in the mean proliferation rates due to stochasticity in mutations (see Fig. S1 - top), so Fig. 3 shows simulation runs with near average behavior. As the tradeoff boundary changes from open to convex to concave, we see natural selection favoring migration over proliferation and a reduction in variation between replicate runs. Contrary to expectation, the convex tradeoff boundary did not produce a generalist phenotype. Instead there is an apparent coexistence of two cell types: one with high migration but moderate proliferation, the other just the opposite. Also, contrary to expectations, a concave tradeoff boundary did not promote the coexistence of extreme phenotypes but instead, natural selection favored higher migration with little to no improvement in proliferation rates.

The sequence of red dots in Fig. 3B show the evolutionary trajectory over time, of the average values, of proliferation and migration rates within the simulated tumor. Each point gives the average phenotype in increments of 5 days until the space is filled. From the spacing of the dots, we see that an open tradeoff boundary produces rapid evolution, rapid space filling, and the highest level of average proliferation rates. A concave tradeoff boundary results in the slowest evolution, slowest space filling, and the lowest average proliferation rate. In going from open to convex to concave tradeoff boundaries, the phenotypes become less proliferative. Thus, they divide, evolve, and fill space more slowly.

### An increased death rate selects for increased proliferation

**Figure 4:**
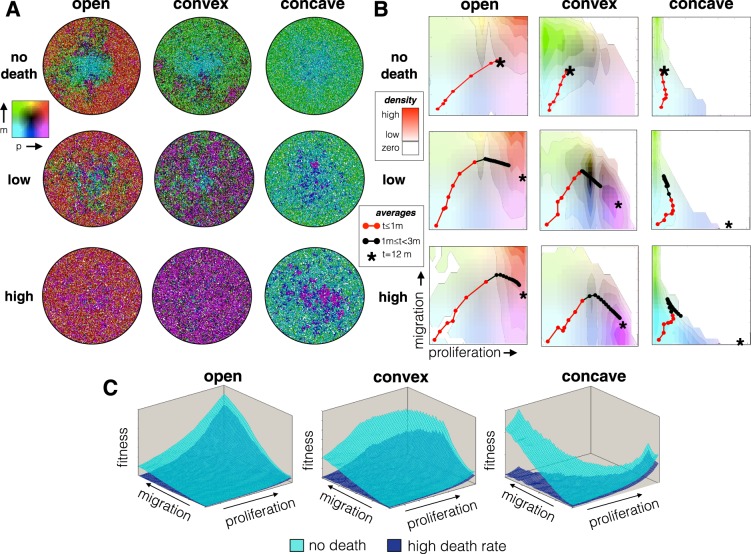
The effects of the death rate (no death, low, and high) and tradeoff boundaries (open, convex and concave) on the evolution of migration and proliferation rates. The probability of death for a single cell is once per week (high death rate) and once every two weeks (low death rate). (**A**) The spatial layout and (**B**) the frequency of trait combinations is shown for a single representative simulation for each case, where the red points and line mark the average trait values every 5 days for the first month. The black points show the continued evolutionary trajectory up until 3 months. The background colors correspond to the density of cell traits at 3 months; Brightly colored areas correspond to high densities, and the completely white area contains no cells. The asterisk shows the average trait values at 12 months. Replicate simulation runs are shown in Fig. S1.

We examined the eco-evolutionary consequences of cell turnover by incorporating random cell death. Figure 4 shows the results when there is no death (top), and a low (middle) and high (bottom) random death rate. The spatial layout is shown to the left, and a density map representing the frequency of trait combinations is shown to the right for the 3-month time point. The evolutionary trajectory of the average trait values the population took for the first 3 months are overlaid on the density map, shown in red, while the black asterisk shows the average phenotype at 12 months.

With non-zero death rates, the phenotypic evolution has two apparent phases: the first occurs while space is relatively sparsely occupied, and the second occurs through cell turnover after the space has filled. During the first phase, phenotypic evolution follows a similar trajectory as the case when there is no death (Fig. 3). However, as space fills, selection favors faster proliferation rates.

When the cells have completely filled the space, the shape of the phenotypic tradeoff boundary (open, convex or concave) strongly influences the endpoint of evolution. Regardless of the death rate, the open boundary favors fast proliferation and high migration speeds. However, as space fills migration speeds matter much less than proliferation rates. Mutational drift in the migration trait leads to a lower mean and higher phenotypic variance than seen in the proliferation trait. Phenotypes with moderate to low proliferation rates become absent with time. With a low death rate, the convex boundary favors the coexistence of a more generalist phenotype with a fast proliferation phenotype. With a low death rate, the convex boundary sees the generalist phenotype outcompeted by fast proliferating cells with lower migration rates. With a low death rate, the concave boundary, as predicted, favors the coexistence between cancer cells with fast proliferation (but low migration) and cells with fast migration (but low proliferation). With a high death rate, the concave boundary favors cancer cells with high proliferation rates at the expense of migration. With no death, replicate runs of the simulation show wide variability in outcomes. Variability between replicate runs becomes greatly reduced when death rates increase (Fig. S1). Comparing the fitness landscapes for each tradeoff for high death rate and no death shows peaks where each of these phenotypes are favored (Fig. 4C).

### Spatially clustered death catastrophes select for migration

**Figure 5:**
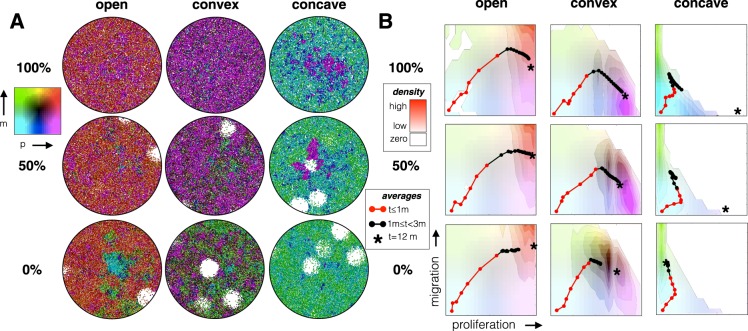
The percent of death that is random vs catastrophic is varied. The top row has 0% catastrophic and 100% random death, the middle row, 50% catastrophic and 50% random death, and the bottom row, 100% catastrophic and 0% random death. The death rate is once per week per cell (same as the high death rate from Fig. 4). (**A**) The spatial layout and (**B**) the frequency of trait combinations is shown for a single representative simulation, where the red points and line mark the average trait values every 5 days for the first month. The black points show the continued evolutionary trajectory up until 3 months. The background colors correspond to the density of cell traits at 3 months; Brightly colored areas correspond to high densities, and the completely white area contains no cells. The asterisk shows the average trait values at 12 months. Replicate simulation runs are shown in Fig. S2.

We introduced significant environmental stochasticity by having all individuals die within a randomly selected 500 μm diameter circular area. This regional catastrophe might represent a sudden (and temporary) loss of blood vasculature, immune cell intrusion, or pooling of toxic metabolites. While keeping the probability of death constant at one death per week per cell (high death rate) we compared three mortality regimes where we varied the fraction of deaths occurring by demographic stochasticity (random cell death) relative to environmental stochasticity (catastrophes). The three regimens had 0%, 50% and 100% catastrophic death. The results are shown in Fig. 5.

In all cases raising the percent of deaths by catastrophes increases selection for migration, and this is consistent across replicate runs (Fig. S2). For the open tradeoff boundary, this results in a similarly high proliferation rate even as the migration rate increases with environmental stochasticity. For the convex tradeoff, there is more variance in phenotypic properties. But, as environmental stochasticity increases, migration is favored over proliferation with a very generalist phenotype emerging when all deaths are catastrophic. For the concave tradeoff boundary, the average phenotype switches from high proliferation and low migration to low proliferation and high migration as environmental stochasticity goes from 0% to 100% of the cause of death. In this case, while there was little variation between simulation runs at 0% and 100%, a greater spread in mean trait values was seen when there was an equal probability of random and catastrophic deaths (Fig. S2 – middle right). The long-term steady state values, however, were consistently toward high proliferation rates and low migration speeds.

## Discussion

Rates of cell turnover matter. As expected, in our model, increasing the death rate speeds the rate of evolution while having little impact on the endpoint of evolution or the equilibrium population size of cancer cells at the end of the simulation (12 months). Our off-lattice model places an upper bound on the space available for cells. While increasing the death rate opens up space, cells fill it quickly as neighboring cells now have the opportunity to successfully proliferate (at even the lowest proliferation rate cells divide once every 50 hours). Longer runs of cell proliferation permit the accumulation of mutations that can increase migration and/or proliferation rates. However, with no deaths, evolution eventually stops on the interior of the tumor and can only occur along the expanding boundary. One sees concentric rings of more highly adapted cells as we move from the center to the edge of the tumor. This is not the case when there is continual cell turnover. While slower in the interior than edge of the tumor, evolution proceeds with the replacement of less fit individuals by those with either higher combined rates of proliferation and migration, or individuals with more successful combinations of traits when the trait-tradeoff boundary has been reached.

The results illustrate the direct impact of cell turnover, throughout the habitable regions, on tumor evolutionary dynamics. However, not all ecological and evolutionary models in the literature incorporate cell death and cell replacement. The distribution of phenotypes among cancer cells in a tumor represent a balance between mutation, drift and selection. With each cell division, mutations can occur that randomly alter proliferation and migration. Those generating higher fitness should increase in frequency, but a large amount of heritable variation is maintained within the tumor due to the stochastic nature of births, deaths and mutations; the lower the rate of cell turnover, the higher the phenotypic variability among cancer cells. In reality, tumors exhibit large amounts of genetic variation – the extent to which this is maintained by mutation and drift and purged through selection remains an open and important question^23,34–38^.

The edge of the tumor likely offers very different conditions in terms of substrates, normal tissue architecture and exposure to the immune system^39,40^. Hence, a number of agent-based models focus on tumor spatial heterogeneity in an environmental context, such as normal cells, stroma, and vasculature^4,5,8–10,41,42^. Here, we considered a much simpler model where all space is equal without regard for the position of blood vessels, and the only factor creating heterogeneity is phenotypic drift during division, which depends on the number and dispersion of cancer cells. The method of inheritance and how drift is imposed could impact the timescales associated with a specific evolutionary trajectory, but this does not affect the steady state values. Furthermore, the model could be extended to include other important tissue interactions such as the influence of vasculature, cell adhesion, and an immune response. These might impact evolution in interesting ways. Other tradeoffs could also be considered, such as proliferation versus survival.

The model aims to specifically address the effects of tradeoffs and cell turnover rates on the speed, trajectory, and endpoint of ecological and evolutionary dynamics within an expanding tumor. As it is, our model has two rather distinct phases starting from a cell with slow proliferation and slow migration. During the first, natural selection favors migration over proliferation as the tumor expands into pristine space, the second favors proliferation over migration once the space has been filled by the cancer cells. This accords with the observation that the edge of tumors may select for more “aggressive” cancer cells defined as those more likely to migrate, invade surrounding tissue, and perhaps initiate metastases^43,44^. If instead, the simulations were initiated with either a fast proliferation rate and slow migration speed or vice versa, the evolutionary endpoint generally remains the same even though the trajectory is different (Fig. S3). There are only a few cases that result in different endpoints from different initial conditions. This happens when i) there is no death, so the space gets filled without achieving the evolutionary endpoint, or ii) there is 100% catastrophic death with a concave tradeoff. For the latter case, when starting with a fast proliferation rate and a slow migration speed the cells remain near the initial phenotype, because even with a more optimal global phenotype (fast migration and slow proliferation) fast proliferation is still selected over the intermediate phenotypes along the trajectory (slow proliferation and slow migration).

There are direct parallels, of our results to ecological systems in which mortality can take the form of the stochastic death of an individual (demographic stochasticity) or the catastrophic death of a group of individuals (environmental stochasticity). In forests, for instance, individual deaths of trees create small gaps in the canopy whereas the blowdown of a group of neighboring trees create large gaps. The size and nature of gaps can result in the slower or faster regeneration of different tree species^45^. Our model considers the eco-evolutionary consequences of different size gaps in the tumor created by either demographic or environmental stochasticity (while holding overall mortality rates constant). As seen in many natural systems, small gaps select for proliferation over dispersal and vice-versa for large gaps^46^. While understudied, temporal variability in local blood flow, immune intrusion, hypoxia, and Ph likely result in varying degrees of local and catastrophic mortality followed by opportunities for recolonization. Histology from biopsies or radiographic imaging of tumors produce a static snapshot that cannot track the fates of individual cells within small regions of a tumor. Our model provides a platform to study how death affects the competition of cells for space and their subsequent evolution.

Tradeoffs between dispersal and survival or fecundity and dispersal are common in natural plant and animal species^47,48^, and several lines of evidence empirically suggest tradeoffs between the traits proliferation versus migration in cancer cells^49–51^. Previous theoretical models have shown how proliferation-migration tradeoffs via phenotypic switching between 2 states can impact tumor growth and evolution^13,28,29^. Tradeoffs in our model are represented by boundaries rather than a switch so that heterogeneity is a spectrum of phenotypes gained on division existing within an allowed trait space. Cancer cells may also experience Allee effects when a critical number of neighboring cells are required for a cell to survive and proliferate^52^. In this case, cell migration from a neighborhood may cause the remaining cells to see a decline in fitness. Our model does not include such an Allee effect. But there is competition for space, and any movement of an individual away from a neighborhood results in a small public good of an increased probability of proliferation to the remaining cells. In the absence of tradeoffs, natural selection should favor improvements in all traits that enhance fitness. As expected in our model, the lack of a tradeoff saw rapid increases in both proliferation and migration. With demographic stochasticity and filled space, migration is no longer under strong selection, so at this point, mutation and drift created a lower mean migration rate with a large phenotypic variance. Generally, a convex tradeoff selected for a generalist phenotype. Under demographic stochasticity this resulted in high phenotypic variance and sometimes the dominance of high proliferation, low migration phenotypes. Environmental stochasticity selected for the more generalist phenotype. A concave phenotypic tradeoff boundary always selected for low proliferation, high migration phenotypes during the tumor’s expansion phase. These were then replaced by high proliferation, low migration phenotypes once the tumor achieved maximum size. The only exception occurred with environmental stochasticity where the high migration phenotype continues to be favored. Experiments that establish the cost of resistance on proliferation of cancer cells often require conditions of strong nutrient limitation^53–56^. In addition, establishing the nature of tradeoffs between migration and proliferation should require the use of invasion/migration assays^57–62^. Such experiments could select for extreme or slow migrators and subsequently observe proliferation rates or select for extreme slow or fast proliferators and subsequently observe migration to determine whether tradeoffs exist.

A number of fitness metrics can be used for mathematically modelling cancer cell population dynamics^63^. These include maximizing some balance of proliferation rates and death rates. In our simulations, the death rate was a constant regardless of phenotype. Hence, natural selection favors phenotypes that maximize the probability of cell division. However, this probability depends not only on the proliferation rate but the probability of having space around the cell to proliferate. Increasing the proliferation rate of a cell directly impacts its fitness. Increasing the migration rate of a cell can indirectly impact tumor fitness by opening up space and keep more neighboring cells cycling, even if they are proliferating more slowly. During the expansion phase of the tumor, available space is relatively large, and the space gained by increasing migration is also very large. Selection will be strong for both proliferation and migration but relatively stronger for migration. When the space in the tumor core is completely filled, both the available space and the fitness advantage gained by increasing migration goes to zero. Hence there is no longer positive selection for migration. By creating many small gaps, demographic stochasticity creates space and thus maintains selection for proliferation, and because the spaces are small, there are no benefits to migration. Environmental stochasticity creates the same amount of total space over time as demographic stochasticity, but this space is more contiguous, so migration is favored as a means of exploiting empty regions. Thus, the fitness advantage gained by migration will be larger and positive when large gaps are present, and there will be positive selection for both proliferative and migratory phenotypes, but a larger selection for migratory phenotypes. Increasing cell turnover directly affects cell fitness, so the indirect tumor fitness gains of increasing migration do not matter as much as individual cell survival so there is positive selection for faster proliferation. Some of these properties will be general to all organisms (e.g., cane toads^64^, and house sparrows spreading in Kenya)^65^, and others just to cancer because it is a densely packed, asexual, and single-celled organism.

Our model has similarities to other models and systems where selection balances two traits. In natural systems this can take the form of seed dispersal versus dormancy in annual plants where the former transports the individual spatially and the latter temporally to less crowded and more favorable places^66,67^. In dispersal-dormancy models, the traits may exhibit tradeoffs via seed size, seed coat thickness, and features that enhance dispersal such as burrs and samara (wings). In cancer, a number of agent-based models consider vector-valued traits. These include degree of glycolytic respiration (Warburg effect) and tolerance to acidic conditions. While not necessarily linked through tradeoffs, the two traits become co-adapted as increased glycolysis promotes acidic conditions necessitating the evolution of acid tolerance^5^. Spatial models often see rings of different trait values extending from the interior to the tumor’s boundary^3,4,6^. In these models, selection happens solely at the tumor edge where there is space to proliferate. In relation to these works our model invites spatially-explicit investigations into how traits evolve in response to population spread, death rates, and demographic versus environmental stochasticity. It emphasizes the critical need to estimate cell turnover via measurements of both death and proliferation rates. A variety of markers and metrics exist for measuring proliferation (e.g. Ki67, mitotic index) and death (e.g. caspases, TUNEL assay). However, these are often just surrogates, rarely measured simultaneously, and generally cannot be measured *in vivo*. Because of these challenges, most data simply describe net tumor growth (i.e. doubling times). We advocate deconstructing this net metric into distinct fractions of proliferating, quiescent, and dying. To study evolving traits such as proliferation-migration tradeoffs, we see a need for non-destructive sensors/markers of cell processes that can be measured through both space and time.

## Methods

We created a simple agent-based model of tumor growth to investigate evolving phenotypes under different constraints. The phenotype is defined as a combination of two traits: the intermitotic time τ, where τ = [10,40] hours, and the migration speed ν, where ν = [0,20] μm/h. The simulation is initialized with one cell in the least aggressive state (τ_max_ = 40 h and ν_min_ = 0 μm/h) centered in a 4 mm circular tissue (Fig. 2A). This cell, and every subsequent cell starts with the specified intermitotic time and counts down at each time step (every minute) until reaching zero. At this point, the cell will split into two daughter cells, keeping one cell at the original position and placing the other 1 diameter away at a random angle. Each daughter cell randomly inherits traits within a range of the parental cell’s traits (τ_daughter_ = τ_mother_ ± (0, 4.5) h and ν_daughter_ = ν_mother_ ± (0, 3) μm/h) as long as it lies within the boundary of traits defined by the tradeoff (Fig. 2B). As long as a cell is not undergoing division during the time step, it will move at its specified speed throughout the 2-dimensional space. The movement of a cell follows a persistent random walk: it will move for a persistence time randomly drawn from a normal distribution p = ${\mathscr{N}}$ (80 min,40 min) and then turn at a random angle before starting over with a new persistence time. The cells can move off-lattice in the 2D space, meaning that they are not confined to reside in a regular gridded structure. A major problem with off-lattice models is that checking for one cell’s interactions amongst all other cells becomes extremely computationally inefficient as the number of cells increase. In order to alleviate this cost, we create a grid (67 μm × 67 μm) that defines neighborhoods within the space. At the top of each frame, all cells are assigned a neighborhood according to their position. Each cell will only check for interactions within a Moore neighborhood of this grid (its current neighborhood as well as its 8 surrounding neighborhoods – see Fig. 2A). If a cell intersects another cell in space it will be assigned a new random direction and a new persistence time. If a cell intersects with the boundary of the space (circle of diameter ~2.7 mm) or is completely surrounded (there is no room for cell division without overlap) it will stop progressing through the cell cycle and stop migrating.

### Bounding the trait space

We limit the possible trait combinations according to i) no tradeoff (open), ii) a convex bound, and iii) a concave bound (see Fig. 2B). Each time a cell divides, new traits are determined giving each option (improve, stay the same, or diminish) the same weight. If the current trait is already on the boundary of trait space, then only options that respect this bound are considered and weighted equally. For the convex case, the forbidden region is created by making a circular arc from the two extreme values where fitness is greatest for each trait but worst for the other (i.e. τ_min_ = 10 h and ν_min_ = 0 μm/h and τ_max_ = 40 h IMT and ν_max_ = 20 μm/h). The trait combinations with the shortest intermitotic times and fastest migration speeds are not allowed. For the concave case, the forbidden region cuts off this space as well, but the circular arc is created using the same points with opposite concavity.

### Cell death

Cell death either occurs randomly distributed or regionally clustered (catastrophic). The probability of death is split between these two types of death with either all random, all catastrophic, or 1:1 mix of random and catastrophic. Random death occurs with a given probability for every cell at every frame. When there are catastrophic death events, all cells within a confined circular region 500 μm in diameter, which is randomly placed, will die. The cells don’t automatically die but wait a randomly chosen period between 6–15 hours before being removed from the system. This is an estimate for how long it takes to go through apoptosis^68,69^.

The probability of death for a single cell is once per week for the high death rate and once every two weeks for the low death rate. The actual death rate is variable because it depends on the number of cells at any time, but when the space is completely full (approximately 13,000 cells), around 2,000 cells are dying per day for the high death rate and 1,000 cells per day for the low death rate.

For the catastrophes, we need to ensure that the number of cells deaths on average is similar to the random death rate, because they happen at a population level at certain time points rather than to individuals. We define the probability of a catastrophic event *p*_cat_ based on the probability of death *p*_death_ and the time intervals *T*_cat_ between catastrophic events:

$${p}_{{\rm{cat}}}=\frac{f{p}_{{\rm{death}}}}{{N}_{{\rm{deaths}}}/N}{T}_{{\rm{cat}}}.$$

Here *f* is the fraction of deaths that are catastrophic, *N*_deaths_ is the number of cells that die from each catastrophic event, and *N* is the total number of cells. Setting the probability of a catastrophic event *p*_cat_ to 1, we can solve for *T*_cat_ to get the appropriate time between catastrophes:

$${T}_{{\rm{cat}}}=\frac{{N}_{{\rm{deaths}}}/N}{f{p}_{{\rm{death}}}}$$

However, because the catastrophic death region will be spaced randomly there is a possibility that a new catastrophe will overlap with an old one before filling back in or will lie on an edge, so in general, there won’t be the same number of cells that die each time a catastrophe occurs. This can be accounted for if the time between events is changed each time based on the number of deaths from the previous event. If the number died previously is less than what would be given by *fp*_death_, then the numerator gets smaller, making a smaller time interval between events, and if the number that died is larger, then the next time interval will be larger. By adjusting after each event, we can compensate for this variation.

### Trait distribution heat maps

For each simulation we show how the 2D combination of traits of all cells are distributed at a specific time point. To create these graphs, we binned the values of intermitotic times and migration speeds for all cells into an 11 × 11 array and used the MATLAB function contour() to define the isolines. Using Pixelmator, each region in the resulting image was converted into white with the value of transparency as a linear gradient of the isoline color so that zero density corresponded to 0% transparent white and maximum density corresponded to 100% transparent white. We overlaid this on our color map to show the densest regions with more of the background color showing through. The average trait values over time made up the top layer of this graph.

### Code availability

Code and interactive website available at https://github.com/jillagal/deathToy/wiki/The-impact-of-proliferation-migration-tradeoffs-on-phenotypic-evolution-in-cancer.

## Supplementary information

Supplementary Information
