# Supplementary material for: The impact of proliferation-migration tradeoffs on phenotypic evolution in cancer
Source: Sci Rep. 2019 Feb 20;9:2425. doi: 10.1038/s41598-019-39636-x (PMC6382810; doi:10.1038/s41598-019-39636-x)
Supplement: Supplementary file 1 — Supplementary Information [file 41598_2019_39636_MOESM1_ESM.pdf]

Supplementary Information

for

The impact of proliferation-migration tradeoffs on phenotypic evolution in cancer

Jill A. Gallaher[1], Joel Brown[1+], and Alexander R. A. Anderson[1*+]

[1]Department of Integrated Mathematical Oncology, H. Lee Moffitt Cancer Center, Tampa, FL USA
*Corresponding author (alexander.anderson@moffitt.org)
[+]These authors contributed equally

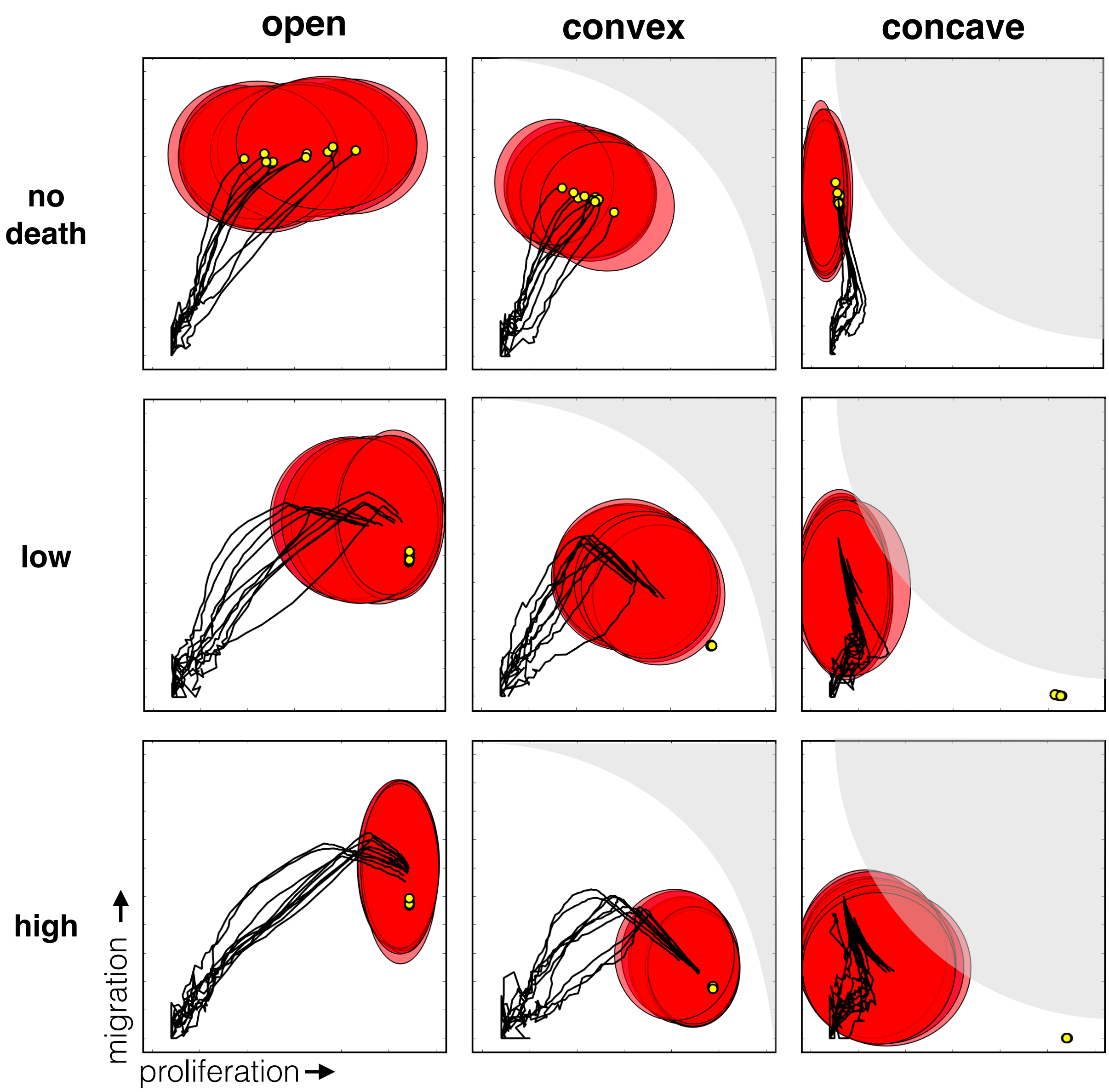

Figure S1. Variation in simulation runs for different death rates, corresponding to results in Figs. 3 and 4 of the main text. Black lines show the trajectories to 100 days from 10 simulations of the mean proliferation rate and mean migration speed. For each simulation, a red ellipse shows the mean traits (center) and standard deviation (radii) for each trait at 100 days. The yellow circles show the mean trait values at 1 year.

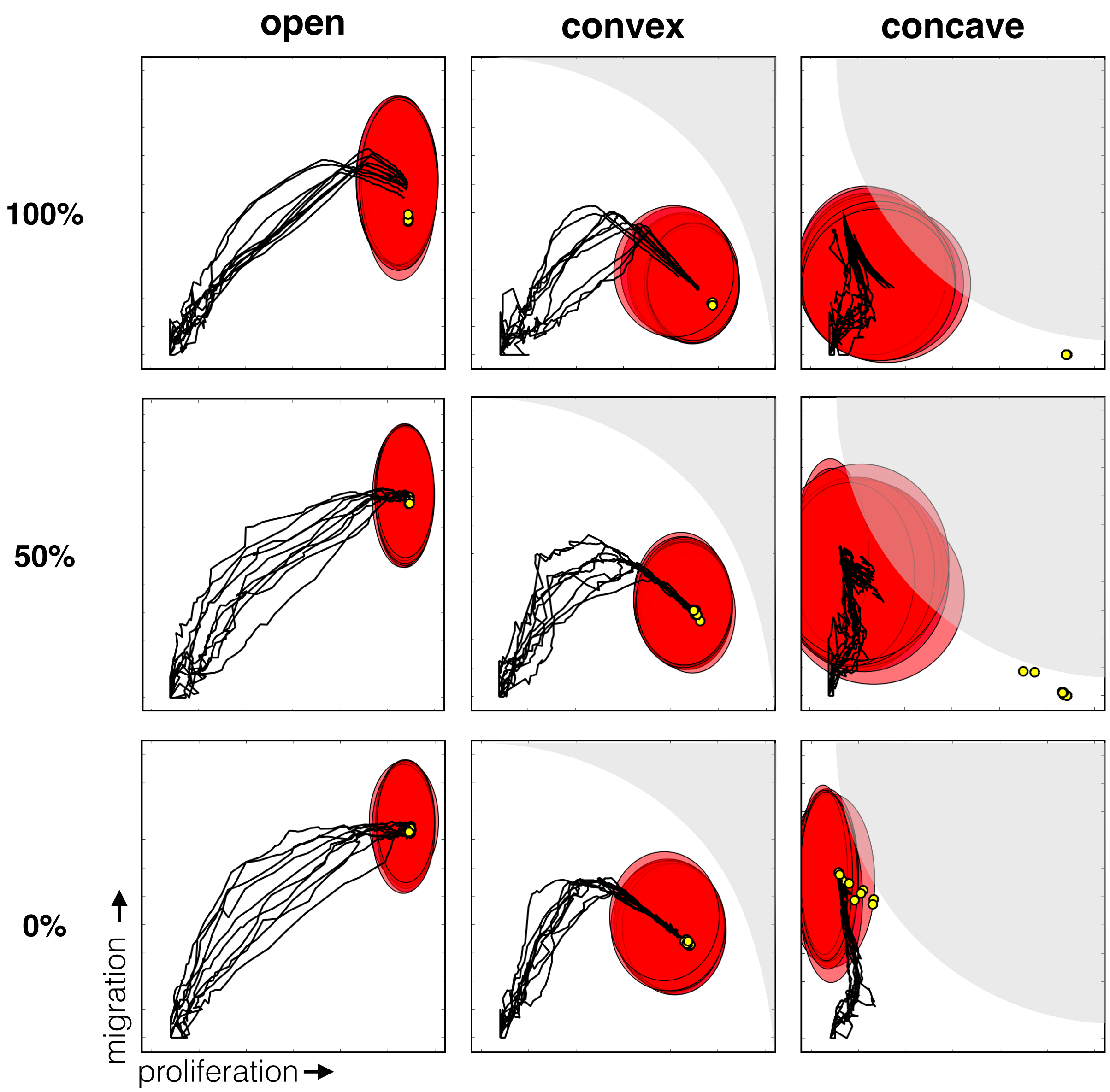

Figure S2. Variation in simulation runs for different percentages of random vs catastrophic deaths, corresponding to results in Fig. 5 of the main text. Black lines show the trajectories to 100 days from 10 simulations of the mean proliferation rate and mean migration speed. For each simulation, a red ellipse shows the mean traits (center) and standard deviation (radii) for each trait at 100 days. The yellow circles show the mean trait values at 1 year.

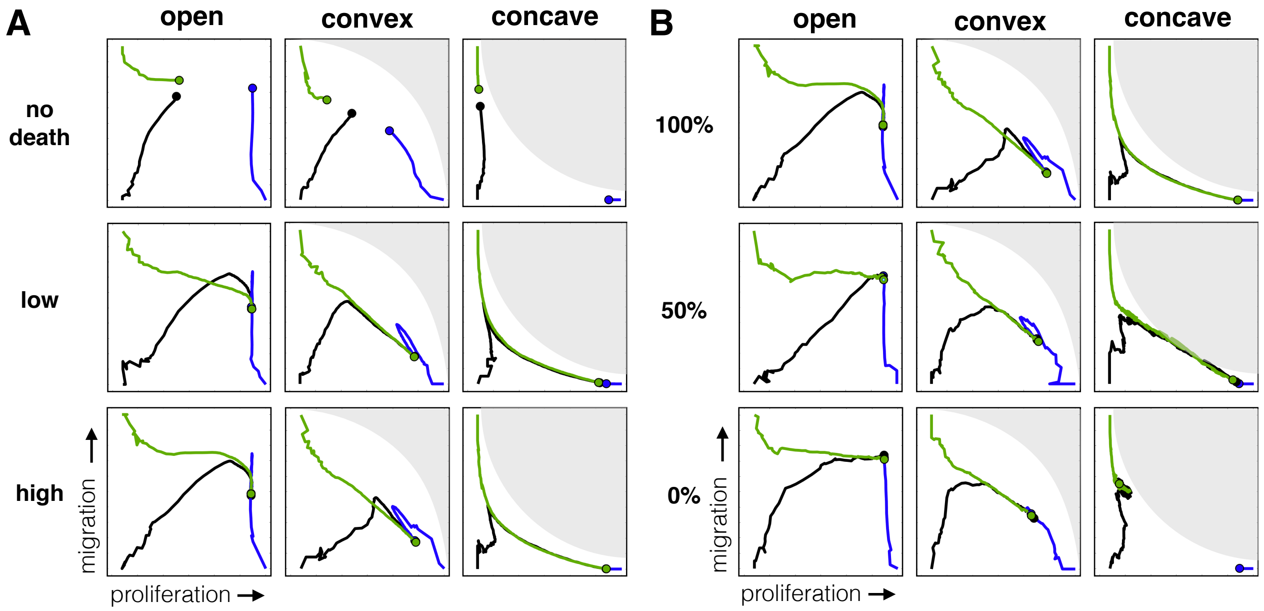

Figure S3. Simulation runs for different tradeoffs with different initial conditions while varying A) the death rate (corresponding to Fig. 4), and B) the percentage of random deaths vs catastrophic deaths (corresponding to Fig. 5). Black lines show a run using the original initial conditions: low proliferation rate, low migration speed. Blue and green lines start with a high proliferation rate and low migration speed and low proliferation rate and high migration speed, respectively. Filled
